# Life satisfaction, resilience and coping mechanisms among medical students during COVID-19

**DOI:** 10.1371/journal.pone.0275319

**Published:** 2022-10-05

**Authors:** Sonia Ijaz Haider, Farhatulain Ahmed, Hassan Pasha, Hadia Pasha, Nudrat Farheen, Muhammad Talha Zahid

**Affiliations:** 1 Centre for Medical Education, School of Medicine, Cardiff University, Cardiff, United Kingdom; 2 College of Medicine and Dentistry, Fatima Memorial College Lahore, Lahore, Pakistan; 3 Bahria University, Karachi, Pakistan; 4 Student Affairs and Services, Aga Khan University, Karachi, Pakistan; 5 Medical College, Aga Khan University, Karachi, Pakistan; St John’s University, UNITED STATES

## Abstract

**Purpose:**

Life satisfaction influences well-being. Medical students often experience more stress as compared to their counterparts in other disciplines as they are required to meet the demands of both academic workload and clinical responsibilities. However, during the current pandemic, in addition to academic changes, inability to complete clinical placements, loss of peer interaction and social connectedness and, deployment to areas in times of crisis could exacerbate their stress. This would impact their ability to cope with stress and eventually influence their life satisfaction. Students approach these challenges in various ways, either positively, religiously, or by avoiding. This study aimed to explore the association between resilience, coping mechanisms and life satisfaction in medical students during the pandemic.

**Methods:**

A cross-sectional web-based survey was conducted from undergraduate medical students from year 1 to year 5. Three instruments were used to measure life satisfaction, resilience, and coping, namely The Brief Resilience Scale, The Satisfaction with Life Scale and the COPE inventory. Mean and standard deviation were calculated for all continuous variables. Robust linear regression model was used for analysis. Hierarchical (forward) stepwise model building technique was used for final model. Alpha cut off was kept at 0.05.

**Results:**

A total of 351 students (out of 500 students) completed the questionnaires. A moderately negative, slightly linear correlation between life satisfaction and avoidant coping was reported. Life satisfaction showed moderately positive, slightly linear correlation with resilience score. Three variables stayed significant in the final model: Resilience, avoidant coping, and religion coping.

**Conclusion:**

Life satisfaction can be improved among medical students by focusing on strategies which enhance resilience. Religion is identified as a significant coping strategy among medical students. Students coping mechanism can vary and more research is needed to assess which types of coping strategies could contribute positively to the quality of their personal and professional lives

## Introduction

Life satisfaction has been defined as “a person’s cognitive and affective evaluations of his or her life” [1]. Life satisfaction strongly influences overall wellbeing. Existing evidence indicate that life satisfaction decreases during medical school among medical students [2, 3]. Some of the consistently reported stressors relate to academics, time pressures, heavy workload, poor relationships, poor student guidance/support finances, fear of failure and examination frequency [4–7]. A recent review described six major themes associated with student distress: adjustment, ethical concerns, exposure to patient death and suffering, student mistreatment, personal life events, and educational debt [8]. Additional evidence indicates that medical students show worse psychological well-being and social relationships than young people in the normative sample [9]. A study which explored stress, resilience and coping in medical students concluded that medical students had higher perceived stress, negative coping, and lower resilience than age and gender-matched peers in the general population [10]. The persistence of previously identified risk factors such as debt burden and clinical phase of school suggests that efforts to curb medical student distress have been inadequate to date [11]. In addition, the medical schools’ environment itself encourages competitiveness rather cooperation among learners. Consequently, it can be inferred that the medical education itself contributes to student distress.

The onset of the pandemic resulted in immediate closure of universities with majority of students forced into an unaccounted learning environment. This became more challenging for medical students as the undergraduate medical curriculum program is structured as preclinical and clinical -where lecture-based teaching was transitioned to an online format, however clinical exposures were not easily replicated [12]. The students were unable to successfully complete clinical placements in a safe and effective manner. Direct patient encounters were replaced with online simulated case scenarios for students. In a recent study, students reported a negative impact of the imposed restrictions on their training, decreased motivation and concentration in an unusual or distraction-prone study environment at home and missing feedback of students and teachers [13]. Moreover, examination restructuring implied that students had to conform to new test format and grading criteria A recent survey of final year medical students in the UK found that over one-third had their objective structured clinical examinations cancelled, with significant effects on self-reported ratings of preparedness to start as doctors [14, 15].

Resilience is the ability to cope mentally or emotionally with a crisis or to return to pre-crisis status quickly [16]. Students can use different coping mechanism for dealing with stressful events. In avoidant coping strategies students change their behavior to avoid about feeling, or doing difficult things [17]. Others can use approach coping by actively focusing on the problematic event or situation [18]. It is not uncommon to use religious coping by using religious beliefs or behaviors to facilitate problem-solving and to prevent or alleviate the negative emotional consequences of stressful life circumstances [19]. Evidence indicates that common positive coping strategies among medical students are respecting one’s limits, setting priorities, avoiding comparisons and participating in leisure activities (cinema, reading, sports, meeting friends and family) [20]. However, during the pandemic, in addition to academic changes, the loss of peer interaction and social connectedness, the possibility of students being deployed to difficult areas in times of crisis, and concerns regarding their personal family well-being were paramount [21]. Considering that under normal circumstances, medical education is challenging for the students, it was expected that the pandemic and the factors resulting from the uncertainty and abrupt changes would exacerbate this issue and affect the overall life satisfaction. Hence, the aim of the study was to explore the relationship and magnitude between resilience, coping orientation and life satisfaction among undergraduate medical students. The study addressed the following research question:

Is there an association between life satisfaction and resilience, and coping orientation among undergraduate medical students during Covid-19?

## Materials and methods

### Participants and procedure

The study involved undergraduate medical students in a private medical college. All the students from year 1 to year 5 were invited to participate in the study via email. The general aim of the study was explained to all the participants. The students completed an online survey using the Google Form platform. Participation in the study was voluntary and all participants provided written informed consent electronically before commencement of the study. The maximum duration for completion of all the questionnaires was 30 minutes. Confidentiality and anonymity were ensured of all the participants data. Participants were given the right to withdraw from the study at any time. The research protocol was approved by the Ethical Committee of the medical college.

### Measures

Demographic characteristics, including age, gender, year of study, marital status, level of education, ethnicity, residence, and previous qualification, was included in the survey. The three measures used in the study were Brief Resilience Scale (BRS), Satisfaction with Life Scale (SWLS) and Coping Orientation to Problems Experienced (COPE) Inventory.

#### Brief Resilience Scale (BRS)

The Brief Resilience Scale consists of 6 items focusing on the ability to recover from stress and adversity. It is on a 5-point Likert scale ranging from 1 = strongly disagree to 2 = strongly agree [22]. The resulting score is a sum of all 6 items. It is on a continuous scale and standard score is reported as a fraction of 5. BRS is reported as a valid and reliable (*α*  = 0.71) measure of resilience [23].

#### The Satisfaction with Life Scale (SWLS)

The Satisfaction with Life Scale developed by Diener, Emmons, Larsen, and Griffin (1985) is a brief measure of life satisfaction (1). It consists of 5 items using a 7-scale scoring system, with 1 = strongly disagree with the statement and 7 = strongly agree. The resulting score is a sum of all 5 items. It is on a continuous scale. The scale is reported to be a valid and reliable (*α*  = 0.80) measure of life satisfaction [24].

Coping Orientation to Problems Experienced Inventory (COPE): The COPE is designed to measure effective and ineffective ways to *cope* with a stressful life event. It consists of 28 items to assess 14 coping strategies on 4-point Likert scale ranging from 1 = strongly disagree to 2 = strongly agree [25]. The tool has four subthemes i.e., avoidant coping, approach coping, humor coping, and religion coping. The scale is reported to be a valid and reliable (*α*  = 0.82) measure of coping strategies [26].

To ensure comparability across results and unification of data collection process, the Brief Resilience Scale (BRS) and The Satisfaction with Life Scale (SWLS) were adapted to 4-point Likert scales ranging from strongly agree to strongly disagree. Decreasing categories of responses did not affect the results because none of the scale items were being excluded or changed. Both the scales report results on continuous data scale, and the same was used for analysis. Therefore, the merging of response categories did not affect the result score variability between students.

### Data analysis

Descriptive analysis was conducted to present the frequency and percentage of the background characteristics of the undergraduate students, including previous education, gender, residence, ethnicity, family system, grade level and leisure activities. Mean and standard deviation (SD) was used to present resilience, life satisfaction and coping orientation scores. The survey was set as such that all questions were marked as “required” thus there was no missing data.

In the multiple linear regression model, satisfaction with life score was entered as the dependent variable. Age, gender, number of siblings, type of residence, family structure, year of medical college, and choice of leisure activities were taken as predictors along with BRS and COPE. Scatterplots were made between all predictor variables vs. life satisfaction score.

Robust linear regression model was used for analysis because the life satisfaction score variable was not normally distributed and could not be transformed. First, univariate linear regression was run between each independent/predictor variable and life satisfaction score, keeping a cut off alpha level of 0.25. Forward stepwise model building technique was used for final model [27]. The predictor variable with highest significance at univariate level were included one by one. All statistical tests were two-sided, and a *p* value< 0.05 was defined as the level of significance. Multicollinearity and interactions (alpha 0.1) were checked. Model fit was checked using residual vs. fitted scatter plot and NP plot of residuals. Analysis was done using STATA 16.0 special edition (Stata Corp, College station, Texas, USA).

### Results

A total of 351 (70%) students (out of total 500 students) completed the questionnaire. There was no missing data. The mean age was 21.5 years. Average number of siblings reported was 3. There was an almost equal representation of students from all 5 years of MBBS. About 86% students had completed FSC before seeking admission in medical college. Students were predominantly females (70%); living with parents (90%); in nuclear family system (82%); and belonged to Punjabi family (77%). About 92% students reported using media to pass their leisure time during COVID-19 pandemic. Studying and learning new skills (17%) and hobbies/creativity (15%) were also reported as leisure activity among students. Demographic characteristics are given in Table 1.

**Table 1:** Demographic characteristics of the undergraduate medical students.

Sr #	Variables	Freq (%)	Mean (SD)
1	Age		21.5 (1.8)
2	Siblings		3.2 (1.6)
3	Previous education		
	Graduate	14 (4.0%)	
	A level	34 (9.7%)	
	Intermediate	303 (86.3%)	
4	Gender		
	Male	105 (29.9%)	
	Female	246 (70.1%)	
5	Residence		
	Living with parents	318 (90.6%)	
	Not living with parents	33 (9.4%)	
6	Ethnicity		
	Punjabi	272 (77.5%)	
	Urdu speaking	59 (16.8%)	
	Others	20 (5.7%)	
7	Family system		
	Nuclear	288 (82.1%)	
	Extended	63 (17.9%)	
8	MBBS		
	Year 1	72 (20.5%)	
	Year 2	65 (18.5%)	
	Year 3	62 (17.7%)	
	Year 4	71 (20.2%)	
	Year 5	81 (23.1%)	
9	Leisure activities		
	Media use	324 (92.3%)	
	Studying, learning skills	59 (16.8%)	
	Hobbies and creativity	52 (14.8%)	
	Family and home chores	19 (0.05%)	
	Physical selfcare	247 (70.3%)	
	Low mood	3(0.01%)	

Table 2 shows the means scores of the life satisfaction, resilience, and coping orientations.

**Table 2:** Means scores of the life satisfaction, resilience, and coping orientations.

Sr. #	Variable	Mean	Std. Dev.	Min	Max
1	Life satisfaction total*	9.1	3.6	0	15
2	Brief Cope				
	Avoidant subtheme	12	6.2	0	29
	Approach subtheme	21.9	6.6	0	36
	Humor subtheme	1.6	1.9	0	6
	Religion subtheme	4.7	1.4	0	6
	Brief Cope total	40.2	11.4	0	68
3	Resilience total	9.3	3.4	1	18
	Standard resilience score	2.6	0.9	0.28	5

* Dependent variable

The total scores on the SWLS ranged from 0 to 15, and the mean score was 9.1 (SD = 3.6). The total scores on the BRS ranged from 1 to 18, and the mean score was 9.3 (SD = 3.4). There was a strong correlation between resilience and life satisfaction. The total scores on the COPE scale ranged from 0 to 68, and the mean score was 40.2 (SD = 11.4). Among the subthemes approach mean score was highest 22 (out of 36), followed by avoidant 12 (out of 36) humor 1.6 (out of 6), and religion 4.7 (out of 6).

Table 3 presents the multivariate robust regression result for the relationship between life satisfaction and the predictor variables.

**Table 3:** Multivariate robust regression model for life satisfaction, resilience, and coping orientations.

Variables	Beta	Std error	P-value	95% CI
Resilience	0.40	0.06	<0.001	0.29, 0.51
Religion	0.59	0.12	<0.001	0.35, 0.82
Avoid	-0.11	0.03	<0.001	-0.17, -0.05

Dependent variable = Life satisfaction score

All independent variables were added at univariate level and the following were significant: resilience score, avoidant coping, religion coping, approach coping, studying and learning skills, and ethnicity. Overall, the model showed a significant association between the independent variables and life satisfaction. Three variables stayed significant in the final model: resilience, avoidant coping, and religion coping. Medical students’ life satisfaction score was predicted by resilience, religious coping, and avoidant coping.

Life satisfaction was positively correlated with religious coping. Life satisfaction showed moderately positive, slightly linear correlation with resilience score. A moderately negative, slightly linear correlation was found between life satisfaction and avoidant coping.

The multivariate model showed that for every 1 unit increase in religious coping score, the life satisfaction score increased by 59%. (p < 0.001), while for every 1 unit increase in total resilience score, the life satisfaction score increased by 40% (p < 0.001), and for every 1 unit increase in avoidant coping score, the life satisfaction score decreased by 11%. (p < 0.001). Approach coping wasn’t a significant mediator of life satisfaction in the model. The demographics of the students who participated in this study were not found to have any significant effect on any of the variables investigated.

## Discussion

The aim of the study was to explore the relationship between life satisfaction, resilience, and coping mechanism among medical students during the pandemic. The findings of the study indicate that there is a difference in the relationship and magnitude between resilience, coping mechanisms and life satisfaction among undergraduate medical students during.

One of the major findings of the present study were that there was a significant correlation between total resilience and life satisfaction, whereby with every one unit increase in resilience, the life satisfaction score increased by 40 percent. A prior research on students in high school reflected similar results whereby life satisfaction was positively related to resilience and positive stress [28]. Multiple studies have reported the favorable effect of resilience on the ability of medical students to deal with stress and to maintain a better quality of life [29] and concluded that resilience among medical students demonstrated a buffering effect on the negative relationship between physical demands and professional quality of life during clerkships. In addition, it was reported that increased resilient behaviours can minimize burnout, as well as cultivate skills needed for promotion of physician resilience and personal fulfillment, and for enhancement of professionalism and patient care [30]. A cross sectional research conducted on Chinese medical students showed that resilience increased life satisfaction whereas perceived stress decreased it [31]. A recent study reported that resilience and life satisfaction play a mediating role in the association between stress and disengagement burnout with resilience exerting greater influence, while in another study, the majority of medical students presented low levels of resilience and high burnout at the time of pandemic. Evidence indicates both internal factors such as optimism, problem, solving, self-regulation, etc. and external factors such as parenting style, family structure, teacher, and peer relations etc. act as buffers or protectors against life stressors [32]. Furthermore, higher resilience in workplace environment is associated with better mental health, reduced stress, and greater well-being [33]. Lack of resilience in students thwarts their emotional and personal development and interferes with their academic progress [34].

Among the various coping approaches investigated in this study, religious coping came out as the most significant predictor of life satisfaction. For every 1 unit increase in religious coping score, the life satisfaction score increased by 59%. These results indicate the value of maintaining faith on a divine entity during uncertain and mostly uncontrollable circumstances. Handling uncertain situations from a religious perspective requires meaningful interpretations of life events and helps develop acceptance for what may come. This is consistent with a recent study [35] which reported lower scores in anxiety and depression due to higher levels of religious coping approach. Similarly in another study, the most effective coping strategy to deal with severe stress was religious activities” as practiced by the majority of the “severely stressed” students [36]. Multiple studies indicate that religiousness encompasses a framework for assigning meaning which is related to reduced stress and the pursuit of mental well-being and life satisfaction [37–39]. Female university students have shown that higher levels of extrinsic religious orientation inversely correlate with depression, anxiety, and stress [40]. Another study conducted to measure the effects of religious coping on hope and wellbeing reported that even when hope is low, wellbeing is found to be high if religious coping is also high [41].

In the present study, avoidant coping showed a modest negative effect on perceived quality of life. For every 1 unit increase in avoidant coping score, the life satisfaction score decreased by 11%. It is reported that students with a high level of stress have a higher preference for avoidance coping strategies [42]. Avoidant coping can have a negative impact on mental health and life satisfaction. It is commonly associated with higher risk of anxiety, and depression over a period of time [43]. Other studies have also demonstrated that avoidant coping leads to higher levels of stress and affects wellbeing in a negative way [44, 45]. A research conducted on adolescent students concluded that avoidant coping led to higher levels of distress and that although other methods of coping do not necessarily predict life satisfaction, avoidant coping always inversely predicts life satisfaction [46]. Lower levels of wellbeing and quality of life have been reported in medical students. In another study exploring perceptions of medical students regarding their quality of life reported scarcity of time for studying, leisure activities, relationships, frustrations with the program and insecurity regarding their professional future as potential inhibitors, whereas factors that increased quality of life were good teachers, classes with good didactic approaches, active learning methodologies, contact with patients, efficient time management and meaningful relationships with family members, friends and teachers [47]. Similarly the perceived quality of life of the Italian medical students was found to be lower than general population and it was suggested to develop resilience among students for improving their quality of life [48]. Yet another comparison of the quality of life between medical students and students of the humanities at the Sarajevo university indicated less satisfaction with overall medical learning experience [49].

Another relevant coping subtheme explored in this study was approach coping. Approach coping wasn’t a significant mediator of life satisfaction in our model. This may be due to the relatively low level of personal control and unpredictability of the situation during COVID-19, whereby a problem-solving approach may not bring satisfactory solutions to stressful life circumstances and in many instances may not even be possible. For example, students who thrive in outdoor activities or social groups would mostly not be able to benefit from approach coping style as they would not have the option to engage in leisure activities that they prefer, thus causing a higher level of frustration and a lower perceived quality of life. Mechanisms for coping with stress and burnout differ among individuals. Mostly these are combination of problem-focused and emotion-focused strategies [50]. In addition, other coping strategies used by students include respecting one’s limits, setting priorities, avoiding comparisons and participating in leisure activities such as cinema, reading, sports, meeting friends and family (20). Time management and self-understanding of learning style are also recommended to minimize the effects of stress among medical students [51].

Several studies emphasize on the importance of undertaking assessment of coping strategies at the beginning of medical education to diagnose a specific trend in physicians’ career development [52, 53]. Evidence indicates that coping strategies of students can change over time with decreased use of active coping strategies and increased use of emotional coping strategies, although emotional strategies were associated with poorer clinical academic performance [54]. This implies that students coping mechanism can vary and it is imperative that students should be orientated to different coping strategies which can enable them to identify those that contribute positively to the quality of their personal and professional lives.

The demographics of the students who participated in this study were not found to have any significant effect on any of the variables investigated. Although there was a greater inclination to use social media as leisure activity, as well as learning new skills and reading, these were unable reach significance level as predictors of quality of life. This finding is consistent with a recent study in which time spent on social media for >4 h increased from 1.1% to 47.72% during lockdown [55], while socializing virtually was reported as 89% and engaging in social media as (85%) [56].

The study had some limitations. The sample in this research comprised of medical students at a single medical college, which prevents conclusions about pattern of coping across the years. The students in the present study were predominantly female (70%), which can impact the generalizability of our findings on general population. Nevertheless, prior research suggests that gender is not a significant correlate of coping styles [57]. All data were obtained through self-reported questionnaires, which could introduce response bias. The participants might have underestimated or overestimated the relationship between the study variables. Although the present study was conducted in a private medical college, there is a likelihood of generalizability of the results to the students in the public medical colleges, considering that religion is a dominant factor within the culture and most sought coping mechanism during stressful times [58].

### Conclusion

This study contributes to a growing body of research on stress and coping among medical students. Results of the present study suggests that life satisfaction can be improved among medical students by focusing on strategies which enhance resilience. Religion has been identified as a significant coping strategy among medical students, hence future studies could focus on interventions to determine its efficacy as a coping mechanism. The presents study adds an important perspective to the debate on how medical students cope with stress in medical school. More research is needed to assess which types of coping strategies could contribute positively to the quality of their personal and professional lives. Future studies could focus on exploring the pattern and impact of different coping strategies on resilience and life satisfaction.
